# Integrated comparative analysis of T-staging of laryngeal carcinoma on parallel versus non-parallel vocal cord CT planes with its potential treatment pitfalls

**DOI:** 10.1371/journal.pone.0330389

**Published:** 2025-09-02

**Authors:** Adeena Khan, Waleed M. S. Fawzy, Syed Shahid Habib, Mamoona Sultan, Manal Bukhari

**Affiliations:** 1 Department of Radiology and Medical Imaging, King Saud University, Riyadh, Kingdom of Saudi Arabia; 2 Department of Physiology, King Saud University, Riyadh, Kingdom of Saudi Arabia; 3 Department of Emergency Medicine, King Saud University, Riyadh, Kingdom of Saudi Arabia; 4 Department of Otolaryngology, Head and Neck Surgery, King Saud University, Riyadh, Kingdom of Saudi Arabia; All India Institute of Medical Sciences, INDIA

## Abstract

Accurate CT staging plays a crucial role in guiding effective management of laryngeal carcinoma, as endoscopic assessment alone may not provide a comprehensive evaluation of tumor spread. Consequences of non-compliance to parallel vocal cord plane for tumors remained unexplored in existing literature. We aimed to compare T-staging parameters of laryngeal carcinoma on CT neck in non-parallel and parallel to vocal cord axial planes and analyse degree of discrepancy between them. We retrospectively studied 34 larynges with squamous cell carcinoma on non-parallel plane① and parallel to vocal cord plane② axial CT scan. Quantitative (anteroposterior AP, transverse Tr, anterior commissure ACom) and qualitative (site, intralaryngeal and extralaryngeal extension) T-staging variables were interpreted in both planes and differences between them were registered. Kappa analysis was employed to get level of disagreement between tumor reporting of each plane. There was minimal to worse agreement between T-staging in these planes (k = −0.059 to 0.353). Difference between means of ACom was significant, but not AP and Tr dimensions. Categorical variables in plane② showed majority of glottic origin (70.5%) and T1a (38.2%) stage, while plane① had subglottic (52.9%) and T2 (41.1%). Overall plane① showed 41.1% altered staging (mostly T1,T2), out of which 71.2% were upstaged. Rest unaffected larynges (28.8%, n = 20) showed either change in origin (41.2%) or ACom involvement (58.8%). Consequently, 73.5% (n = 25) patients in plane ① manifested at least one change, so can jeopardise targeted management. Results established that non-adherence to reporting on parallel CT larynx can result in significant variability in principal qualitative and quantitative T-stage tumor parameters, hence imperiling patients to over or undertreatment.

## Introduction

Larynx is the commonest location in aerodigestive tract cancers and contributes around 25–30% of all head and neck tumor cases with mortality rate of up to 33.9%. Over the past three decades despite global decrease (around 1%) in incidence and mortality rate of laryngeal cancers, there is still rising trend in some developing countries and Asia carries the most burden share. Majority of them are squamous cell carcinoma (98%) [1,2].

Laryngologists primarily rely on CT (computed tomography) scan to stage laryngeal carcinoma (LC). Unlike CT, laryngoscopy has limitations to evaluate salient features of tumor including depth of invasion, involvement of paraglottic fat (PGF), pre-epiglottic (PEF), laryngeal cartilages and extra laryngeal tissues which are main indicators of T-staging [3]. So, imaging report has a decisive role in selection between multifarious therapies like radiotherapy (RT), chemotherapy, targeted therapy, laser surgery, partial or total laryngectomy and combination treatments [4,5]. T-stage assessed on CT images has 82.6% accuracy and majority of inaccuracy observed is in T2 stage tumors [6].

CT interpretation of pathological larynx confronts various challenges because of its dynamicity, complex morphology, proximity of three subdivisions (supraglottis SpG, glottis G, subglottis SG) and obliquity of vocal cord (VC) [7,8]. For staging of cross- sectional imaging to be in accord with pathological extension requires CT axial plane parallel to vocal cord (PVC) which can be attained either by pre-selection on scout image or multiplanar reformation (MPR) [9,10]. PVC plane and dynamic CT have found to significantly alter normal laryngeal parameters and tumor staging respectively [9,11]. Non-parallel VC (NPVC) plane shows falsified glottic anatomy, can change tumor staging or its localization because it passes obliquely through VC, i.e., from anterosuperior to posteroinferior direction at various degree of angulations [9,12,13].

Precision in imaging description of structures involved in LC is an essential requirement for optimal management plan. Isolated endoscopy has staging accuracy of 51.3%, but if combined with CT scan it improves up to 80.2% [14,15]. Monotherapy is usually curable in early staged carcinoma, while combined therapy warrants multimodal therapy [16]. Overstaging or understaging is one the greatest pitfalls in LC management failure and can lead to adverse outcome [12]. According to an update on American Society of Clinical Oncology Clinical Practice Guideline for laryngeal preservation strategies to treat LC, it is recommended to initially treat T1 and T2 stage with laser endoscopic resection or RT and avoid combination therapy. Locally advanced T2 G and SpG tumors show better outcome with primary open surgical resection. Preferences of locally advanced T3/ T4 disease are laryngeal preservation surgery, combined chemoradiotherapy or isolated RT. Favourable outcome of extensive T3 or large T4a tumors can be achieved with total laryngectomy and postoperative radiotherapy [17,18]. Thickening (>2.1 mm) of anterior commisure (ACom) is also one of the major prognostic indicators and considered to commute success rate of non-surgical treatment [19].

Various causes of erroneous staging have been described [12,20]. Our recent research has associated importance of PVC plane in defining precise anatomy and quantitative parameters of a normal glottis with concerns about its sequel in laryngeal pathologies [9]. However, any study seeking the role of PVC tomographic plane in staging LC is not yet available. Therefore, we had a notion to carry out T-staging of LC on both NPVC and PVC planes along with investigation of consensus between them which may practically improve imaging stage, therapeutics and their outcome. Description of various tumor parameters in this study like upstaging, downstaging, alteration in origin or invasion might be useful not only for radiologists to deliver reporting of LC more meticulously but also laryngologist to be more particular in their management decision.

## Materials and methods

### Patient selection

Time of collection of data was between 6^th^ May 2021 till 6^th^ May 2022. Biopsy proven laryngeal squamous cell carcinoma patients available between 1^st^ July 2018 and 30^th^ June 2022 were included in this retrospective study after approval from our institutional review board (Project No. E-21-5875 & Ref No. 21/0428/IRB). Consent was not taken as this was a retrospective study which was fully anonymised in images and data set. Contrast enhanced CT scan (CECT) neck of these patients were further inspected for their inclusion and exclusion criteria. Children (≤ 18 years), missing data, CT neck without contrast, suboptimal technique (slice thickness >3mm, adducted vocal cord, artifacts, inappropriate patient position), post-surgical, post-radiation larynx and secondary involvement or mass effect by tumour of other neck structures were excluded from our list.

### CT imaging

Images were acquired on GE (General Electric) CT scanner (GE revolution CT 256 multidetector). Scan was done in supine with neck in neutral or slightly extended position. Patient was allowed to continue quite breathing during examination and forbidden to hold breath, take deep breath, talk or swallow. Due to lack of facility for preselection of VC plane in our scanner, initial scanning was performed in plane perpendicular to CT table. 60 mL intravenous contrast (omnipaque 300-lohexol) was infusion at the rate of 3 millilitre/second with 45 seconds delay. Scan geometry included FOV (field of view) 25 cm, pitch 0.8 and 512 × 512 pixel matrix using 120 kVp (kilovoltage peak), automated tube current and 0.5-second rotation time. Scanned head and neck area was from base of skull till arch of aorta. Axial 0.625 mm helical images with 1–3 mm reconstruction in plane parallel to VC was performed. Both unformatted (NPVC) and reformatted (PVC) images were sent to picture archiving and communication system.

### Image interpretation

Two radiologists who were blinded to histological staging of LC and having experience in head and neck radiology examined CT scans. Images were interpreted on soft tissue algorithm except for identification of laryngeal cartilages and bony involvement. Anterior neck length (aNL) was taken in sagittal plane. Parameters for laryngeal mass were analysed on both unformatted (NPVC, plane①) and reformatted (PVC, plane②) planes. True axial plane② reformation was attained by positioning posterior axial plane marker on cricoarytenoid joint (CAJ) as a standard for localisation of glottic level, followed by anterior adjustment of this plane by tilting it parallel to VC accordingly.

### Statistical analysis

Statistical analysis was performed using SPSS (IBM SPSS Statistics for Windows, Version 201.0 Armonk, NY: IBM Corp.). Descriptive data was expressed as mean ± standard error (SE) along with range (minimum and maximum). Qualitative data was exhibited as frequencies and percentages. Kolmogorov-Smirnov and ShapiroWilk tests were performed for normality.

To determine degree of agreement or disagreement in the interpretation and staging of squamous cell carcinomas related to larynx we performed kappa analysis. Kappa analysis was interpreted as per Cohn’s kappa scale range which is from less than 0 up to 1. Values were comprehended as follows: ≤ 0 no or agreement by change, 0.01–0.2 slight, 0.21–0.4 fair, 0.41–0.6 moderate, 0.61–0.8 substantial and 0.81–1.00 almost perfect agreement between readings of two planes.

## Results

### Subject characteristics and analysis

**Fig 1:**
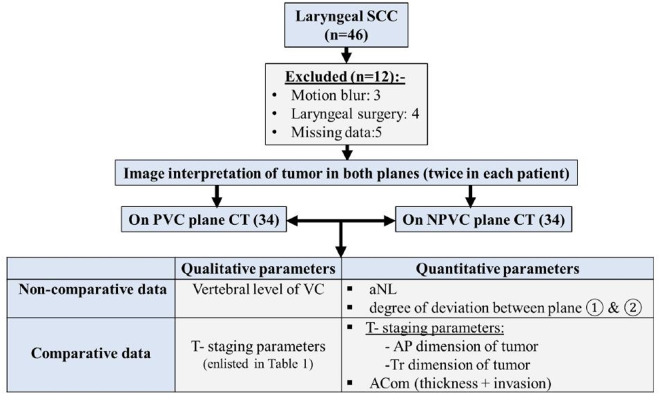
Flowchart summarising patient selection and study variables. n = number of patients, SCC = squamous cell carcinoma, PVC = parallel to vocal cord, NPVC = non-parallel to vocal cord, VC = vocal cord, aNL = anterior neck length, AP = anteroposterior, Tr = transverse, ACom = anterior commisure.

Patients with histological proven laryngeal squamous cell carcinoma were 46 in number. Out of these 34 patients (male = 29, female = 5) fulfilled our selection criteria. How patients were selected and what tumor parameters were recorded in study are summarized in flow chart (Fig 1, Table 1). Patient body characteristics are tabulated in Table 2. Tumor parameters were extracted on the basis of staging and radiological anatomical features of each laryngeal level [8,21]. T-stage was assigned according to American Joint Committee on Cancer (AJCC) system of staging laryngeal cancers (2018) [22].

**Table 1:** Tumor parameters studied for T-staging in parallel and non-parallel vocal cord axial CT scan planes of larynx.

Qualitative T-staging parameters recorded in axial CT plane
Origin (according to tumor bulk and anatomical landmark of each laryngeal subdivision) Side (unilateral, bilateral or contralateral extension) Extension in *SpG*: false cord fat, aryepiglottic folds, epiglottis, PGF, PEF Extension in *glottis:* unilateral or contralateral VC involvement, ACom, PCom, cricoarytenoid joint, upper part of cricoid Extension in *SG:* till cricoid or below, cricoid cartilage Thyroid cartilage (inner, outer cortex involvement) Extralaryngeal tissues (muscles, vessels, thyroid gland, hypopharynx)

SpG = supraglottis, PGF = paraglottic fat, PEF = pre-epiglottic fat, VC = vocal cord, ACom = anterior commisure, PCom = posterior commisure, SG = subglottis.

**Table 2:** Selected patients’ build and demographic data.

n = 34	Mean ± SD	Range
**Age (yrs)**	55.5 ± 14.2	19-78
**Height (cm)**	163.9 ± 10.40	130-178.6
**Weight (kg)**	74.6 ± 22.58	22-125.8
**BMI (kg/m** ^**2**^ **)**	27.37 ± 6.94	13.02-42.33
**aNL (cm)**	66.9 ± 22.61	26.6-122

n = number of patients, SD = standard deviation, yrs = years, BMI = body mass index, aNL = anterior neck length.

### Comparison of laryngeal tumor parameters in CT plane ① and ②

#### Tumor origin.

On comparing frequency of tumor origin in each plane it was noted that in plane① majority of LC were SG in origin (n = 18, 52.9%) while in parallel plane② were arising mostly from glottis (n = 24, 70.5%). Overall, 17 (50%) patients showed disparity in origin. All of them appeared to be arising one laryngeal subdivision distal to their actual point of origin due to obliquity of an axial plane, i.e., supraglottic or glottic/ACom tumor in plane② was seen as glottic or subglottic tumor in plane① respectively. Tumors of glottic location were affected the most (14/24, 58.3%) followed by ACom and SpG (Table 3).

**Table 3:** Frequency distribution of tumor location in each axial CT plane of larynx, NPVC plane① and PVC plane②.

Origin (n = 34)	NPVC plane ①%(n)	PVC plane②%(n)	Difference of %(n) between plane①②
**Supraglottis**	8.8 (3)	14.7 (5)	5.9 (2)
**Glottis**	35.3 (12)	70.5 (24)	35.2 (12)
**ACom**	2.9 (1)	5.8 (2)	2.9 (1)
**Subglottis**	52.9 (18)	8.8 (3)	44.1 (15)

n = number of patients, NPVC = non-parallel vocal cord, PVC = parallel vocal cord, ACom = anterior commissure.

#### Dimensions.

Means of all tumor dimensions were compared between NPVC plane① and PVC plane② and showed that average AP (13.29 ± 7.8 and 13.12 ± 8.4 mm ± SD) and Tr (9.59 ± 7.8 and 9.07 ± 7.9 mm ± SD) dimensions of tumor were statistically insignificant (p = 0.749 and 0.137), respectively. Unlike these, ACom showed significant (p = 0.000) difference between their mean values of planes, i.e., 9.76 ± 6.6 and 4.77 ± 4.4 mm ± SD. Means of difference between AP, Tr and ACom in these planes were 2.3 ± 1.8, 1.36 ± 1.5 and 6.6 ± 5.3 mm ± SD respectively.

#### T-staging.

**Fig 2:**
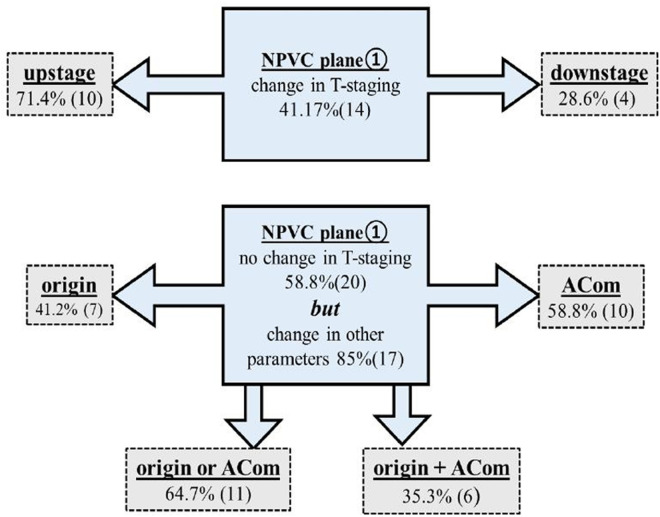
Details of altered laryngeal tumor parameters in axial non-parallel vocal cord plane of CT larynx. NPVC = non-parallel vocal cord, ACom = anterior commissure.

Frequency distribution of T-staging showed that majority of patients in plane① were of T2 stage (n = 14), while T1a (n = 13) in plane②. Maximum difference in staging frequency between these planes was observed in T1a (20.6%) followed by T2 (14.7%) and T1 (5.9%). Overall, 14 patients (out of 34) showed change in staging and were predominantly upstaged. Majority of altered staging was in early LC (T1a, T2) (n = 13, 38.2%) and only one was advanced (T3). Out of 20 unaffected larynges 17 patients showed alteration in at least one of the major tumor parameters, i.e., either origin, ACom thickness or combination of them. Consolidation of data established that there were overall 25 (73.5%) CT scans which were affected by NPVC axial plane in terms of either T-stage, origin or ACom. Elaborated data pertaining to staging is demonstrated in Table 4 and Fig 2.

**Table 4:** Frequency distribution of T-staging in each axial CT plane of larynx, NPVC plane① and PVC plane②.

T-staging (n = 34)	NPVC plane①% (n)	PVC plane② % (n)	Difference in % (n) between planes①②
**T1a**	17.6(6)	38.2 (13)	20.6 (7)
**T1**	11.7(4)	5.8 (2)	5.9 (2)
**T2**	41.1(14)	26.4(9)	14.7 (5)
**T3**	11.7(4)	11.7(4)	0 (0)
**T4**	17.6(6)	17.6(6)	0 (0)

n = number of patients, NPVC = non-parallel vocal cord, PVC = parallel vocal cord.

#### Deviated plane angle and tumor parameters.

Due to obliquity of axial plane, 79.4% (n = 27) of plane① crossed more than one vertebral level (anterosuperior to posteroinferior) rather parallel to vertebral body or disc plane.

Average angle of deviation between plane ① and ② was 20.9° ± 7.83. Patients were grouped into two ranges of angle deviation, group I (≤ 20°) and group II (> 20°). Majority (n = 19, 55.8%) of them were from group I (≤20°, n = 19) with maximum deviation of axis to about 42.5° (range = 8.5-42.5°). Frequency distribution of T-staging in both groups II, II showed T2 and T1a as majority in both planes① and ② (Table 5). To sum up, 25 (73.5%) out of 34 larynges potentially altered management decision keeping T-stage, origin and ACom as primary determinants of LC.

**Table 5:** Distribution of T-staging in each axial CT plane of larynx, NPVC plane ①and PVC plane ②, according to deviated range of axis measured as angles (≤20° and >20°).

Angles	Group I ≤ 20°		Group II > 20°	
T-stage	NPVC plane① % (n)	PVC plane② % (n)	NPVC plane① % (n)	PVC plane② % (n)
**T1a**	15.8 (3)	36.8 (7)	20 (3)	40 (6)
**T1**	5.3 (1)	5.3 (1)	20 (3)	6.7 (1)
**T2**	52.6 (10)	31.6 (6)	26.7 (4)	20 (3)
**T3**	5.3 (1)	5.3 (1)	20 (3)	20 (3)
**T4**	21 (4)	21.0 (4)	13.3 (2)	13.3 (2)

N = number of patients, NPVC = non-parallel vocal cord, PVC = parallel vocal cord.

**Fig 3:**
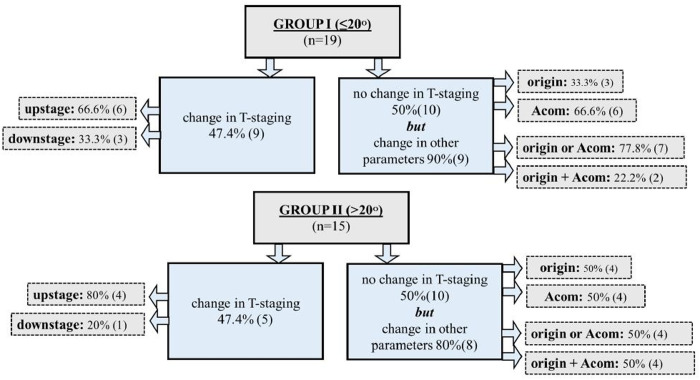
Elaborated data of altered laryngeal tumor parameters in axial non-parallel vocal cord plane① of CT larynx, according to deviated range of axis (angles) i.e., ≤20° and>20°. NPVC = non-parallel vocal cord, PVC = parallel vocal cord, ACom = anterior commissure.

Group I patients showed most variation in tumor parameters. On scrutinising data set of plane①, group I (n = 19) versus group II (n = 15) CT scans which showed change in stage were 9 (47.4%) versus 5 (33.3%), for origin were 8 (42.1%) versus 9 (60%) and ACom status of tumor as 7 (36.8%) versus 5 (33.3%) respectively (Fig 3).

### Kappa analysis for agreement between unformatted CT plane① and reformatted plane②:

Reformatted PVC plane② CT scan being considered ideal to interpret laryngeal pathologies, so unformatted plane① CT was compared with it through kappa (k) analysis to quantify agreement between imaging findings observed in these planes. It was noted that advanced LC showed only change in stage of one patient, so only early staged LC were analysed.

Overall, kappa results suggested that there was significantly high variability ranging from slight to worse agreement between plane① and ② in staging early carcinoma accurately indicating less reliability of NPVC plane in determination of appropriate treatment for LC. Negative kappa coefficient (k) signifies level of agreement is worse than chance. Standard error of mean (SE) was also low for all of them indicating precision of study data set. Out of early-stage LC, T1 stage showed worst (−0.059) agreement between two CT planes in staging the disease. Obtained k (SE) values for T1a, T1 and T2 were 0.206 (0.106), −0.059 (0.069) and −0.147 (0.113) respectively. In terms of location, SG tumors showed most discordant results (negative k value). Biases related to differences in imaging were not affected by the agreement as both planes were assessed on same patient having no difference in CT protocol (Table 6).

**Table 6:** Results of kappa analysis depicting level of agreement between reporting of laryngeal tumor origin and its staging in parallel and non-parallel CT axial planes.

T-stage				
	Kappa value	SE of kappa	Observed agreement (n = 34) (%)	95% CI (range)
**T1a**	0.206	0.106	60.2	−0.003 to 0.415
**T1**	−0.059	0.069	47.0	−0.193 to 0.076
**T2**	−0.147	0.113	42.6	−0.370 to 0.075
**Origin**				
**SpG**	0.059	0.078	52.9	−0.094 to 0.212
**Glottic**	0.353	0.113	67.6	0.131 to 0.575
**ACom**	0.029	0.050	51.4	−0.068 to 0.127
**SG**	−0.441	0.101	27.9	−0.638 to −0.244

N=number of patients, SE= standard error of mean, SpG=supraglottis, ACom=anterior commissure, SG=subglottis.

## Discussion

Our study investigated effect of unformatted NPVC plane on T-staging and quantitative parameters of LC by taking parameters of PVC plane as a standard. Reporting on an inaccurate axial plane led to T-stage alteration in considerable (2/5^th^) number of patients and more than 2/3^rd^ of patients showed change in at least one variable (T-stage, origin or ACom). Additionally, we found an overall poor level of agreement between stage reporting of two planes. Accuracy of imaging modalities, dynamic and static laryngeal CT for tumor staging have been a subject of discussion in past, but significance of plane accuracy in LC and its other surgically important variables have never been evaluated before [11,23,24].

LC is one of the commonest tumors in otolaryngology and 7 times more common in male than female population [25]. Our sample also had 6 times more males than females [26].

**Fig 4:**
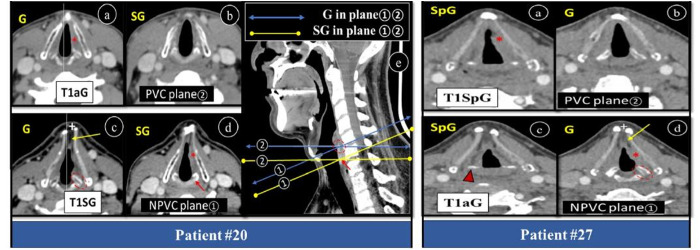
Axial and sagittal post-contrast CT larynx showing change in T1 tumor origin due to CT plane. **Patient # 20:** Axial (a-d) and sagittal (e) post-contrast CT larynx showing change in location of T1aG tumor PVC plane to T1SG in NPVC plane. Plane② (a,b) showing left VC origin, while plane① (c,d) left SG origin. Asterisks (*) showing areas of tumor extension (G, SG), dotted oval (c,e) is CAJ at glottic level, arrow is at cricoid cartilage of SG. Sagittal plane (e) is pictorial demonstration of reason for tumor location change. **Patient #27:** Axial post-contrast CT larynx showing change in location of T1 SpG in PVC plane to T1aG in NPVC plane. Plane② (a,b) showing left false cord origin, while plane① (c,d) left true VC origin. Asterisks (*) showing areas of tumor extension (SpG, G), arrowhead is upper part of AC of SpG level, dotted oval is CAJ at glottic level. Also note false anatomical landmarks of G in NPVC plane, i.e., fat (long yellow arrow) and open thyroid notch (+) in both patients. *SpG = supraglottis, G = glottis, NPVC = non-parallel vocal cord, PVC = parallel vocal cord, AC = arytenoid cartilage, CAJ = cricoarytenoid joint.*

Although tumor origin is primarily examined on laryngoscopy, but cross-sectional scan is mandatory if tumor is subtle, equivocal and distal to VC [10,12,27]. Primary location of tumor especially SpG versus G is very crucial as unlike G, SpG has propensity towards occult nodal metastasis and surgeons may consider morbid neck dissection as well [18]. Treatment of SG tumor is more aggressive than other sites which includes total laryngectomy, total or partial thyroidectomy, neck dissection and post-surgical RT at tumor site [12,21]. Tumor origin or its base can be a matter of confusion on CT scan but there are some diagnostic clues which help to assess tumor origin like bulk of tumor and laryngeal anatomical landmarks. SpG is identified by vocal fat density, glottis by CAJ and SG by a cricoid ring [8,28]. Using the above criteria in our study, true plane② had 70.5% glottic followed by 14.7% SpG, 8.8% SG and 5.8% ACom tumors which matches literature data of 60% glottis, 35% SpG and remaining either from SG or overlaps between three laryngeal subdivisions [26]. In contrast, plane① showed SG origin as majority (52.9%). Most altered origin in plane① was glottis and showed almost 50% low frequency compared to true glottic plane (35.3% versus 70.5%) and literature (35.3% versus 60%). The reason for glottic tumor mimicking SG tumor was because anterior part of axial plane passed through a glottic tumor, but its posterior non-parallel part is more oblique and cut through cricoid ring instead of CA joint making actual glottic tumor as SG in location (Fig 4, patients#20,27).

Additionally, compared to other laryngeal divisions less depth of glottis or VC do not allow much deviation of axial plane from true VC plane. Another feature of NPVC is that it also passes obliquely through multiple vertebral levels in majority 87.4% and this is comparable to our frequency of 79.4% [9]. On analysis of isolated change in tumor origin according to degree of plane deviation in group I and II (≤20°,  > 20°) it showed similar results, 8 versus 9 patients. Next affected locations after glottis were ACom and SpG which changed to SG in 50% (1 of 2 patients) and glottic in 40% (2 of 5 patients) respectively (Fig 4, patient #27). None of SG tumors showed any down step of location (tracheal origin) which was probably due to only 3 patients of SG origin or its larger craniocaudal dimension. Above inconsistent data for tumor origin in NPVC plane is at risk of mismanagement in terms of pre-therapeutic planning and surgical strategy.

**Fig 5:**
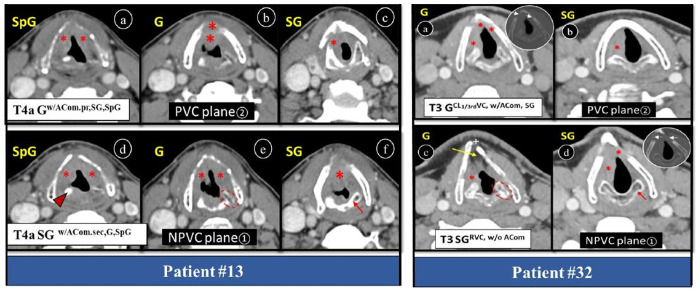
Axial post-contrast CT larynx showing change in origin (considering tumor bulk) and extension of T4a stage glottic tumor due to CT plane. **Patient#13:** Plane② (a-c) showing ACom origin glottic tumor, while plane① **(d-f)** SG origin. Asterisks (*) showing areas of tumor extension (SpG, G, ACom, SG, TC), arrowhead is upper part of AC at SpG level, dotted oval is CAJ at glottic level, arrow is SG cricoid cartilage. **Patient#32:** Plane② (a, b) showing right glottic tumor extending to ACom, CL < 1/3rd of VC and SG, while plane① (c, d) showing right SG tumor extending to ipsilateral G without ACom and CL VC extension. Asterisks (*) showing areas of tumor extension (G, CL G, ACom, SG), dotted oval is CAJ at glottic level, short arrow is SG cricoid cartilage. Also note false anatomical landmarks of G in NPVC plane, i.e., fat (yellow long arrow) and open thyroid notch (+). *SpG = supraglottis, G = glottis, SG = subglottis, ACom = anterior commissure, w/ACom.pr=with primary origin from anterior commissure, w/ACom.sec=with secondary invasion of anterior commissure, NPVC = non-parallel vocal cord, PVC = parallel vocal cord, AC = arytenoid cartilage, CAJ = cricoarytenoid joint, w/,w/o=with and without, RVC = right vocal cord, CL = contralateral.*

Correct identification of ACom thickness or involvement is decisive between voice preservation and compromise surgical decision. These tumors are at risk of spread to VC, cartilage, PEF, PGF, and lymph nodes because here Broyles tendon is attached to thyroid lamina devoid of perichondrium [12,28,29]. Extension to it may compromises prognosis and is a potential for recurrence after voice preserving trans-oral CO_2_ laser microsurgery (TOLS). In some cases, it also precludes partial laryngectomy [29–31]. Once a SpG tumors extends to ACom, patient will no longer a candidate for SpG laryngectomy because 2–3 mm tumor free margin is required to perform voice sparing surgery. Early glottic tumor with ACom infiltration has better local control if treated primarily with surgery (open or laser) than RT [5,12]. Primary isolated ACom tumor is rare and they show good outcome with RT alone [5]. We had only two cases (patient #13, 15) in our sample which showed tumor bulk in ACom. One out of them became SG tumor in plane① because of visibility of bulk of tumor at the level of cricoid instead of CAJ (Fig 5, patient#13).

**Fig 6:**
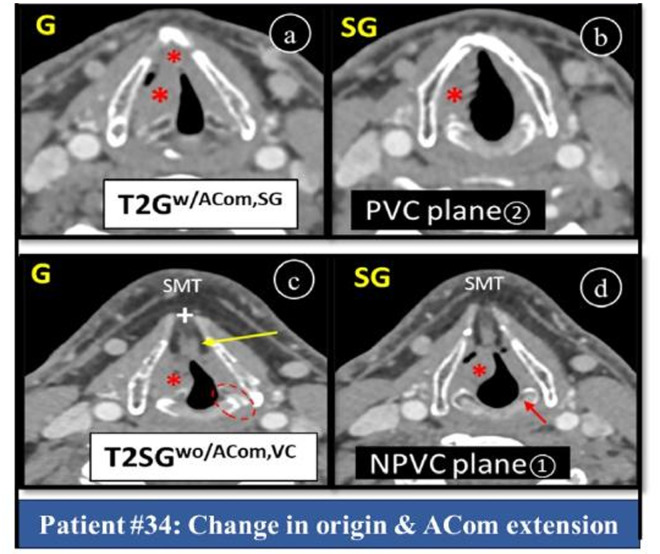
Axial post-contrast CT larynx showing change in origin and ACom involvement due to CT plane. **Patient#34:** Plane② (a, b) showing ACom invasion, while plane① (c, d) showing normal falsely thickened ACom having fat (long arrow). Asterisks (*) showing areas of tumor extension (G, ACom, SG), dotted oval is CAJ at glottic level, short arrow is SG cricoid cartilage. (+) shows open thyroid notch. Note false anatomical landmarks of G in NPVC plane, i.e., SMT, fat and open thyroid notch. *SpG = supraglottis, G = glottis, w/, w/o ACom=with, without anterior commissure invasion, SG = subglottis, NPVC = non-parallel vocal cord, PVC = parallel vocal cord, SMT = submental triangle, CAJ = cricoarytenoid joint, VC = vocal cord.*

Regardless of staging, 10 of our patients showed ACom invasion in parallel plane which dropped to 2 in non-parallel plane risking patients to undertreatment and unfavourable outcome (Fig 6, patient#34). Disease in posterior commissure excludes supracricoid laryngectomy and tumor is at risk for spread for spread into hypopharynx. Our study did not show any posterior commisure as an additional extension in plane① [12,21].

With reference to our previous study on normal larynx, NPVC CT plane demonstrated abnormal ACom thickening (pseudo thickening having fat) in 97.9% cases with statistically significant difference (9.76 mm) between means of two planes [9]. It is 5.8 mm in our current study and includes both type of patients having truly involved (with soft tissue density, patient# 13) and falsely thickened (uninvolved with fat density, patient# 34) ACom.

Tumor volume is a key predictor in treatment of early LC with definitive RT. Supraglottic tumors <6–8 ml and T3 glottic <2.5 ml are considered as cut offs for favourable outcome. Volume >2.5 ml is considered for surgical management [12,32,33]. Reduction in need of salvage total laryngectomies and comparable oncological outcome can be obtained with transoral laser microsurgery (TLM). Tumor dimensions are also taken into prime consideration in TLM as here tumor is removed in multiple blocks rather en block and finally sent for pathological analysis. Adequate margin for glottis is about 1–2 mm and up to 5–10 mm in SpG [17,34]. Tumor volume calculation was out of the scope of our study aims, but AP and Tr dimensions were taken which indirectly reflect volume (see all figures). Results did not show up significant difference between planes which might be either due to small sample or major effect of CT plane on tumor staging only.

Staging accuracy especially T-stage is prime requirement for management selection in LC due to availability of various voice conserving treatments. But pertinent therapy is not very specific for each stage because many factors are involved in decision which include age, comorbidities, patient priorities, local expertise and rehabilitation services [20,35]. T- staging has a very thin discriminatory line between each stage which can make significant changes in surgeon choice. Discriminator between T1a-T1b glottic is contralateral involvement, T1-T2 is transglottic spread or impaired cord mobility, T2-T3 is cord fixation or paraglottic space or inner cartilage involvement and T3-T4 is outer cartilage or extralaryngeal tissues involvement [22]. Summarised T-staging of AJCC is tabulated (Table 7).

**Table 7:** Tabulated summary of T-staging of laryngeal carcinoma in each laryngeal subdivision. Asterisks (*) means presence of any one feature.

Stage	Supraglottis	Glottis	Subglottis
**T1**	1 part of SpG	T1a = unilateral, T1b = bilateral	Limited to SG
**T2**	• > 1 part of SpG • ± glottis with normal movement	• SpG or SG* • impaired VC mobility	• + VC • ± impaired VC mobility
**T3**	• immobile VC* • PGF, PEF • post-cricoid region • inner cortex TC	• immobile VC* • PGF • inner TC	• immobile VC* • PGF • inner TC
**T4a**	• outer cortex TC • adjacent structures* (TG, oesophagus, tongue, neck muscles, trachea)		
**T4b**	• invasion into vertebral muscle • carotid artery involvement* • mediastinal structures		

SpG = supraglottis, VC = vocal cord, PEF = pre-epiglottic fat, PGF = paraglottic fat, TC = thyroid cartilage, TG = thyroid gland.

SpG carcinomas can be inaccurately staged on CT examination in 25% of cases and misinterpretation of VC or ACom involvement was one of them. Here it was attributed to observers’ variation, but we should also consider non-parallel axial CT plane as its potential cause [9,36]. Audit of 38 laryngeal squamous cell carcinomas demonstrated 45% over staging and 10% under staging by CT scan. Here majority were upstaged from T3 to T4 due to cartilage involvement but unlike our study staging was compared with pathology [20]. Another study found more overstaging in stage T2 [6]. We observed 71.4% overstaging and 28.5% undernstaging due to untrue CT plane and majority in early LC.

**Fig 7:**
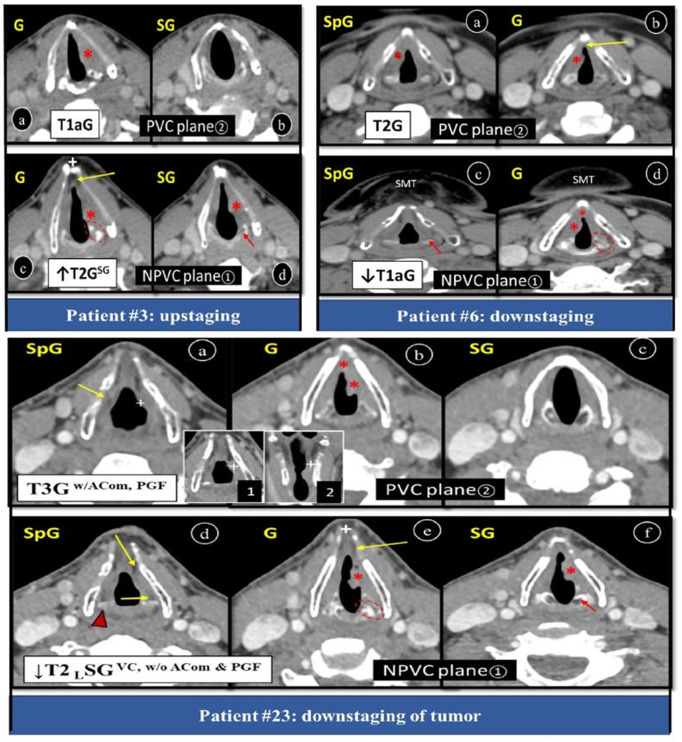
Axial post-contrast CT larynx showing change in T-staging of LC due to CT plane. **Patient#3:** Plane② (a, b) showing localised left glottic tumor, while plane① (c, d) showing glottic tumor extending to SG. Asterisks (*) showing areas of tumor extension (G, SG), dotted oval is CAJ at glottic level, short arrow is SG cricoid cartilage, long arrow is fat in ACom, (+) is thyroid notch, ↑ is upstage. **Patient#6:** Plane② (a, b) showing right glottic tumor extending to SpG, while plane① (c, d) showing localised right glottic tumor with ACom extension only. Asterisks (*) showing areas of tumor extension (G, ACom, SG), dotted oval is CAJ at glottic level, short arrow is SpG AC, ↓ is downstage. **Patient#23:** Showing change in T-staging, location, PGF and ACom involvement due to CT plane. Plane② (a, b) showing left glottic tumor extending to SpG with minor PGF involvement, while plane① (c, d) showing bulk in SG with G extension and normal PGF. Asterisks (*) showing areas of tumor extension (PGF, G, SG), dotted oval is CAJ at glottic level, arrowhead id upper part of AC in SpG, red arrow is SG cricoid cartilage, all yellow arrows are fat, ↓ is downstage, (+) is thyroid notch. Note false anatomical landmarks of G in NPVC plane, i.e., fat, SMT and open thyroid notch. *G = glottis, SpG = supraglottis, SG = subglottis, ACom = anterior commissure, NPVC = non-parallel vocal cord, PVC = parallel vocal cord, AC = arytenoid cartilage, CAJ = cricoarytenoid joint, SMT = submental triangle, ACom = anterior commisure, PGF = paraglottic fat.*

T1 and T2 tumors if not so deep can be treated with external RT depicting 90% and 75% success rate respectively with good voice control and regardless of ACom involvement. Spread of tumor to laryngeal ventricles or arytenoid precludes partial supraglottic laryngectomy. T1a glottic tumor without ACom involvement evidenced to get benefit from voice preservation therapies like RT and TOLS or open partial laryngectomy. We found that all 10 upstaged tumors belonged to T1a glottis out of which 6 were upstaged to T2 SG and 4 to T1 SG carcinoma but none to SpG, thus excluding voice securing options (Fig 7, patient #3). Out of 4 downstaged tumors, 3 were downgraded from T2 to T1a glottic and one from T3 to T2 glottic tumor, hence risking patient to tumor recurrence and poor control (Fig 7, patient# 6). None of downstaging was due to change in location of tumor. T1, T2 tumor with ACom infiltration and extension to contralateral less than 1/3^rd^ of VC is an indication for vertical hemilaryngectomy. If it is not so deep, then RT is also an option [5,7,10,27,31,37]. We had only one glottic tumor having unilateral disease with contralateral 1/3^rd^ VC extension in plane② which was changed to unilateral disease in plane① leaving patient at risk of erroneous tumor remedy (Fig 5, patient# 32).

T2 staging is incomplete without assessment of VC mobility which is not possible on CT scan. T2 with normal VC mobility respond well to laser resection. VC impairment declines local control after laser treatment, so supracricoid partial laryngectomy is better selection in these cases [5]. Out of 4 only one patient of T3 stage in our study had fixed VC.

SG tumor usually demands aggressive treatment. If extension to SG is > 1 cm or cricoid is involved, it is an indication for total laryngectomy [10,12,21,38]. Out of 18 patients which showed SG origin in plane①, 50% (9) showed alteration in T-staging (7 up, 2 down).

T3 LC usually treated with supracricoid laryngectomy which is also an alternative surgery for T4a as well, only in specific patients. For selected patients with variable laryngeal and extralaryngeal tissue invasion, concurrent chemoradiotherapy (CRT), induction chemotherapy and hyper-fractionated or accelerated RT provide high level of organ preservation compared to RT alone. However, better survival rate is with total laryngectomy along with RT compared to other therapies [5].

In our research other important management modifiers like PEF, cartilage invasion or extralaryngeal spread were not influenced by NPVC plane axial CT scanning. Invasion of PGF contraindicates TOLS, so distinction between minor, major, inferior and superior PGF invasion is very necessary to choose between open partial horizontal laryngectomy versus total laryngectomy [3,7]. We encountered only one patient whose staging was downgraded from T3 to T2 due to PGF invasion in plane② to normal PGF in plane①, but none was overstaged due to PGF invasion (Fig 7, patient# 23).

Laterality of tumor was also remained unaffected except one patient which changed from contralateral VC involvement to unilateral VC (patient#32). These finding could be treatment limiters. Findings in laryngeal tumor which risk failure to non-surgical or voice conservative therapies are listed in Table 8.

**Table 8:** List of laryngeal regions contributing to prediction of failure to less morbid treatments.

T-stage parameters as predictor of local treatment failure in LC
PGF and PEF involvement*
Acom invasion*
PCom involvement
Tumor volume*
Location (subglottic extension)*
VC mobility
Cartilage invasion
Hypopharyngeal extension
Extralaryngeal spread

PGF = paraglottic fat, PEF = pre-epiglottic fats, ACom = anterior commissure, PCom = posterior commisure, VC = vocal cord.

Note: asterisk* marked were the parameters which were affected by NPVC plane in our study.

This project has some limitations. T-staging on imaging planes was not compared with histopathology. Alongside practical implication by data correlation with surgical decision and its outcome in upstaged and downstaged tumors by plane① is also not included which could have greater impact, but this can be a future directive. Due to retrospective data we had some technical constraints including different phases of respiration, phonation and variable slice thickness of up to 3 mm which were challenge especially in small sized tumors. As there was no comparative study discerning significance of true VC axial plane in staging LC, we could not substantiate our study by similar evidence. Study was conducted on limited sample size and need to be tested on larger cohorts. Patient wise tabulated representation of our data including staging in both planes, interpretation of changes in T-stage as well its extension are also indexed in our manuscript for better understanding (Table 9).

**Table 9:** Elaborated representation of whole sample of laryngeal carcinomas along with track changes in T-staging and extension of tumor.

Patient no.	Tumor stage with location		Effect of plane①NPVC		
	Plane②PVC	Plane①NPVC	Origin	T-stage	Extension
1.	T1a _L_G	T2 _L_G ^SG^	–	**↑** T1a→T2	SG
2.	T3 _L_G ^fVC^	T3 _L_G ^fVC^	–	–	–
3.	T1a_ L_G	T2_ L_G ^SG^	–	↑ T1a→T2	SG
4.	T4a _BL_SpG	T4a _BL_SpG	–	–	–
5.	T2 _R_G ^FC, w/ACom^	T2 _R_SG ^VC, w/o ACom^	G to SGꜜ	–	SG, × ACom
**6.**	T2_ R_G^ FC^	T1a_ R_G^ w/ACom^	–	T2→T1a↓	× FC, ACom
7.	T1a_ L_G	T1_ L_SG	G to SGꜜ	↑ T1a→T1	–
8.	T1a_ L_G	T2_ L_SG^VC^	G to SGꜜ	↑ T1a→T2	SG
9.	T4a _R_G	T4a _R_G	–	–	–
10.	T1a_ L_G	T1_ L_SG	G to SGꜜ	↑ T1a→T1	–
11.	T1a _R_G	T1a _R_G	–	–	–
12.	T1a_ L_G	T2_ L_SG^VC^	G to SGꜜ	↑ T1a→T2	SG
13.	T4a _BL_G^w/ACom.pr^	T4a_BL_SG ^w/ACom.sec,VC^	G^ACom.pr^ to SGꜜ	–	–
14.	T1a _R_G	T1a _R_G	–	–	–
15.	T2 _BL_G ^w/ACom.pr, VC, FC^	T2 _BL_G ^w/ACom.pri, VC, SG^	–	–	×FC, SG
16.	T1a_ L_G	T1_ L_SG ^VC^	G to SGꜜ	↑ T1a→T2	SG
17.	T2 _R_G ^w/ACom, rVC^	T2 _R_SG ^w/o ACom^	G to SGꜜ	–	×ACom
18.	T4a _R_SpG	T4a _R_SpG	–	–	–
19.	T1a _L_G	T1a _L_G	–	–	–
20.	T1a_ L_G	T1_ L_SG	G to SGꜜ	↑ T1a→T1	–
21.	T4a _L_SG	T4a _L_SG	–	–	–
22.	T1 _L_SpG	T1a _L_G	SpG to Gꜜ	–	–
23.	T3_L_G^ w/ACom, PGF^	T2_ L_SG^ VC, w/o ACom & PGF^	G to SGꜜ	T3→T2↓	×PGF, × ACom, SG
24.	T1a_R_G^w/ACom^	T1_ R_SG^ w/o ACom^	G to SGꜜ	↑ T1a→T1	×ACom
25.	T2 _L_G^FC^	T2 _L_G^FC^	–	–	–
26.	T2_ L_G ^w/ACom, SG^	T1a_ L_G^ w/o ACom^	–	T2→T1a↓	×ACom, × SG
27.	T1_L_SpG	T1a _L_G	SpG to Gꜜ	–	–
28.	T1a_ R_G	T2_ R_SG^VC^	G to SGꜜ	↑ T1a→T2	SG
29.	T3 _L_SG	T3 _L_SG	–	–	–
30.	T3 _L_SpG	T3 _L_SpG	–	–	–
31.	T2_ R_G^ SG^	T1_ R_SG	G to SGꜜ	T2→T1↓	–
32.	T3_R_G^CL1/3rdVC, w/ACom, SG^	T3_R_SG^RVC, w/o ACom^	G to SGꜜ	–	×CL1/3^rd^VC, × ACom
33.	T3 _L_SG ^w/ACom^	T3 _L_SG ^w/o ACom^	–	–	×ACom
34.	T2 _R_G^w/ACom, SG^	T2 _R_SG^VC, w/o ACom^	G to SGꜜ	–	×ACom

Boxes colored red indicate upstaged and blue downstaged patients.

NPVC = non-parallel vocal cord, PVC = parallel vocal cord R = right, L = left, BL = bilateral, CL = contralateral, G = glottis, SpG = supraglottis, SG = subglottis, ACom = anterior commisure, ACom.pri = anterior commisure as primary location, ACom.sec = anterior commisure as secondary location, VC = vocal cord, fVC = fixed vocal cord, rVC = restricted vocal cord movement, PGF = paraglottic fat, FC = false cord, w/= with, w/o= without; ↑= upstage, ↓= downstage, ꜜ = distal shift, **- **= no change, ×= not invaded.

## Conclusion

There was significant disparity between staging and other variables of early-stage LC in PVC and NPVC axial CT plane hence questioning authenticity of imaging data in NPVC plane. Majority of patients showed at least one or more than one dissimilarity in tumor parameters in the form of T-staging, location of tumor or status of ACom. This mandates reformatted plane PVC to avoid injudicious laryngectomies due to upstaging and local control failure due to downstaging. It situates substantial contribution and responsibility of radiologists and laryngologists to verify accuracy of CT plane before taking any management decision for patients with LC.

## Supporting information

S1 File Larynx staging supplemental data. (XLSX)
